# (*E*)-3-(3,5-Dimeth­oxy­phen­yl)-1-(2-meth­oxy­phen­yl)prop-2-en-1-one

**DOI:** 10.1107/S1600536813006302

**Published:** 2013-03-09

**Authors:** Yoongho Lim, Dongsoo Koh

**Affiliations:** a Division of Bioscience and Biotechnology, BMIC, Konkuk University, Seoul 143-701, Republic of Korea; b Department of Applied Chemistry, Dongduk Women’s University, Seoul 136-714, Republic of Korea

## Abstract

In the title mol­ecule, C_18_H_18_O_4_, the dihedral angle between the benzene rings is 52.52 (7)°. The C=C bond of the central enone group adopts a *trans* conformation. The relative conformation of the two double bonds in the enone group is *s*-*transoid*. In the crystal, mol­ecules are linked by pairs of weak C—H⋯O hydrogen bonds, forming inversion dimers.

## Related literature

For the synthesis and biological properties of chalcone derivatives, see: Shin *et al.* (2012 ▶); Hwang *et al.* (2011 ▶). For related structures, see: Fun *et al.* (2012 ▶); Lee *et al.* (2012 ▶); Prasath *et al.* (2010 ▶).
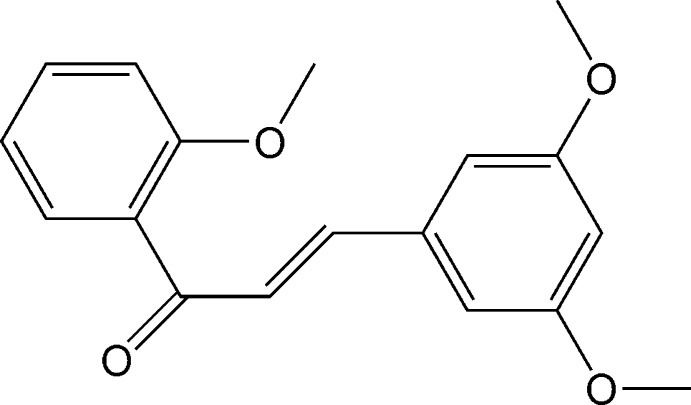


## Experimental

### 

#### Crystal data

C_18_H_18_O_4_
*M* *_r_* = 298.32
Monoclinic, 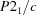

*a* = 12.0925 (18) Å
*b* = 8.4460 (12) Å
*c* = 15.109 (2) Å
β = 92.340 (3)°
*V* = 1541.9 (4) Å^3^
*Z* = 4
Mo *K*α radiation
μ = 0.09 mm^−1^
*T* = 200 K
0.24 × 0.14 × 0.10 mm

#### Data collection

Bruker SMART CCD diffractometer
11328 measured reflections
3865 independent reflections
1544 reflections with *I* > 2σ(*I*)
*R*_int_ = 0.053

#### Refinement

*R*[*F* ^2^ > 2σ(*F* ^2^)] = 0.043
*wR*(*F* ^2^) = 0.132
*S* = 0.81
3865 reflections
202 parameters
H-atom parameters constrained
Δρ_max_ = 0.21 e Å^−3^
Δρ_min_ = −0.27 e Å^−3^

### 

Data collection: *SMART* (Bruker, 2000 ▶); cell refinement: *SAINT* (Bruker, 2000 ▶); data reduction: *SAINT*; program(s) used to solve structure: *SHELXS97* (Sheldrick, 2008 ▶); program(s) used to refine structure: *SHELXL97* (Sheldrick, 2008 ▶); molecular graphics: *PLATON* (Spek, 2009 ▶); software used to prepare material for publication: *SHELXTL* (Sheldrick, 2008 ▶).

## Supplementary Material

Crystal structure: contains datablock(s) I, global. DOI: 10.1107/S1600536813006302/lh5589sup1.cif

Structure factors: contains datablock(s) I. DOI: 10.1107/S1600536813006302/lh5589Isup2.hkl

Supplementary material file. DOI: 10.1107/S1600536813006302/lh5589Isup3.cml

Additional supplementary materials:  crystallographic information; 3D view; checkCIF report

## Figures and Tables

**Table 1:** Hydrogen-bond geometry (Å, °)

*D*—H⋯*A*	*D*—H	H⋯*A*	*D*⋯*A*	*D*—H⋯*A*
C2—H2⋯O1^i^	0.95	2.51	3.457 (3)	172

Symmetry code: (i) 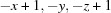.

**Fig. 1.**
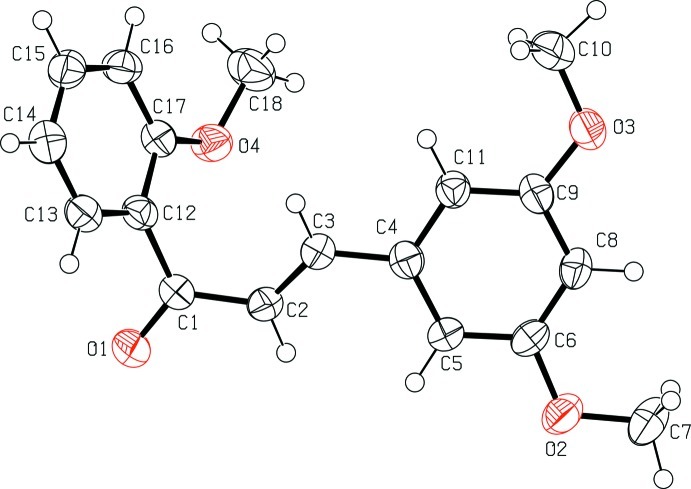
The molecular structure of the title compound, showing displacement ellipsoids drawn at the 50% probability level.

**Fig. 2.**
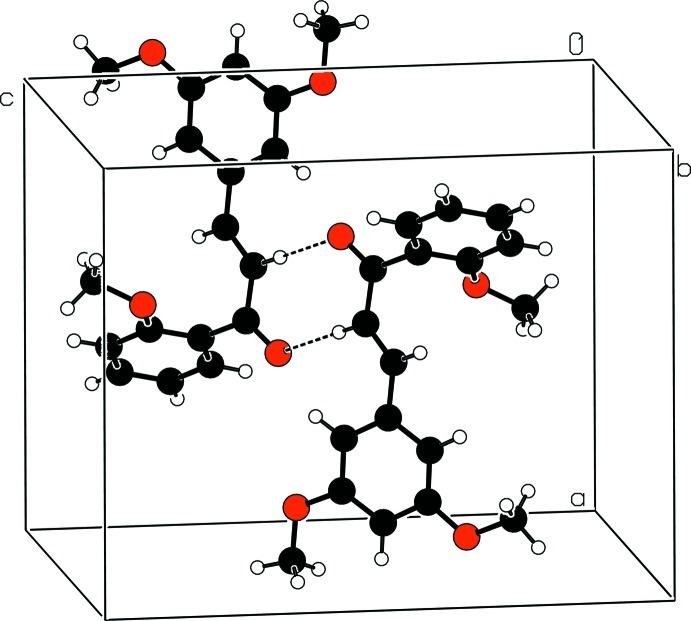
Part of the crystal structure showing an inversion dimer formed via a pair of weak intermolecular C—H···O hydrogen bonds shown as dashed lines.

## supplementary crystallographic information

### Comment

Chalcones have an α,β-unsaturated carbonyl (enone) group which connects two aromatic rings at the 1,3-positions. Typically, the conformation of enone system is s-*cisoid*, in which the C═C and C═O double bonds are *cis* with respect to each other. Few examples of s-*transoid* conformations have been reported in the literature (Fun *et al.*, 2012; Prasath *et al.*, 2010). As a part of our studies on the substituent effects of chalcones on structures and biological activities (Shin *et al.*, 2012; Hwang *et al.*, 2011), the crystal structure of title compound has been determined.

The molecular structure of the title compound is shown in Fig. 1. The relative conformation of two double bonds of the central enone group is s-*transoid*. The *trans* configuration at the C1═C2 bond is reflected in the O1-C1-C2-C3 torsion angle of -168.7 (2)° compared to the value of -1.1 (5)° in a structure with an s-*cisoid* configuration (Lee *et al.*, 2012). The dihedral angle between the benzene rings is 52.52 (7)°. Two methoxy groups at *meta* positions of the C4-C6/C8/C9/C11 ring are essentially co-planar with the ring [C8—C6—O2—C7 = -2.4 (3)° and C11—C9—O3—C10 = -1.2 (3)°]. However, the methoxy group at the *ortho* position of the C12-C17 ring is slightly twisted with respect to the benzene ring [C16—C17—O4—C18 = 21.6 (3)°]. In the crystal, molecules are linked by a pair of weak C—H···O hydrogen bonds to form inversion dimers (Table 1, Fig. 2).

### Experimental

To a solution of 3,5-dimethoxybenzaldehyde (415 mg, 2.5 mmol) in 30 ml of ethanol was added 2-methoxyacetophenone (300 mg, 2 mmol) and the temperature was adjusted to around 276 K in an ice-bath. To the cooled reaction mixture was added 2 ml of 50% aqueous KOH solution, and the reaction mixture was stirred at room temperature for 5 h. This mixture was poured into iced water (50 ml) was acidified (pH = 3) with 3 N HCl solution to give a precipitate. Filtration and washing with water afforded crude solid of the title compound (560 mg, 94%). Recrystallization of the solid in ethanol gave single crystals (mp: 353–355 K).

### Refinement

H atoms were placed in calculated positions and refined as riding with C—H = 0.95–0.98 Å, and *U*iso(H) = 1.2 *U*eq(C) or *U*iso(H) = 1.5 *U*eq(C_methyl_).

### Crystal data

**Table:**

C_18_H_18_O_4_	*F*(000) = 632
*M**_r_* = 298.32	*D*_x_ = 1.285 Mg m^−^^3^
Monoclinic, *P*2_1_/*c*	Mo *K*α radiation, λ = 0.71073 Å
Hall symbol: -P 2ybc	Cell parameters from 2539 reflections
*a* = 12.0925 (18) Å	θ = 2.7–28.1°
*b* = 8.4460 (12) Å	µ = 0.09 mm^−^^1^
*c* = 15.109 (2) Å	*T* = 200 K
β = 92.340 (3)°	Block, colorless
*V* = 1541.9 (4) Å^3^	0.24 × 0.14 × 0.10 mm
*Z* = 4	

### Data collection

**Table:**

Bruker SMART CCD diffractometer	1544 reflections with *I* > 2σ(*I*)
Radiation source: fine-focus sealed tube	*R*_int_ = 0.053
Graphite monochromator	θ_max_ = 28.5°, θ_min_ = 1.7°
φ and ω scans	*h* = −16→14
11328 measured reflections	*k* = −11→10
3865 independent reflections	*l* = −20→19

### Refinement

**Table:**

Refinement on *F*^2^	Primary atom site location: structure-invariant direct methods
Least-squares matrix: full	Secondary atom site location: difference Fourier map
*R*[*F*^2^ > 2σ(*F*^2^)] = 0.043	Hydrogen site location: inferred from neighbouring sites
*wR*(*F*^2^) = 0.132	H-atom parameters constrained
*S* = 0.81	*w* = 1/[σ^2^(*F*_o_^2^) + (0.0575*P*)^2^] where *P* = (*F*_o_^2^ + 2*F*_c_^2^)/3
3865 reflections	(Δ/σ)_max_ < 0.001
202 parameters	Δρ_max_ = 0.21 e Å^−^^3^
0 restraints	Δρ_min_ = −0.27 e Å^−^^3^

### Special details

**Table:**

Geometry. All esds (except the esd in the dihedral angle between two l.s. planes) are estimated using the full covariance matrix. The cell esds are taken into account individually in the estimation of esds in distances, angles and torsion angles; correlations between esds in cell parameters are only used when they are defined by crystal symmetry. An approximate (isotropic) treatment of cell esds is used for estimating esds involving l.s. planes.
Refinement. Refinement of *F*^2^ against ALL reflections. The weighted *R*-factor *wR* and goodness of fit *S* are based on *F*^2^, conventional *R*-factors *R* are based on *F*, with *F* set to zero for negative *F*^2^. The threshold expression of *F*^2^ > σ(*F*^2^) is used only for calculating *R*-factors(gt) *etc*. and is not relevant to the choice of reflections for refinement. *R*-factors based on *F*^2^ are statistically about twice as large as those based on *F*, and *R*- factors based on ALL data will be even larger.

### Fractional atomic coordinates and isotropic or equivalent isotropic displacement parameters (Å^2^)

**Table:**

	*x*	*y*	*z*	*U*_iso_*/*U*_eq_	
C1	0.40664 (18)	0.2012 (2)	0.41632 (14)	0.0370 (5)	
O1	0.35034 (13)	0.11209 (19)	0.46017 (11)	0.0569 (5)	
C2	0.52596 (18)	0.2050 (2)	0.43027 (13)	0.0366 (5)	
H2	0.5597	0.1242	0.4658	0.044*	
C3	0.59125 (17)	0.3144 (2)	0.39646 (13)	0.0359 (5)	
H3	0.5557	0.3937	0.3609	0.043*	
C4	0.71093 (17)	0.3259 (2)	0.40791 (14)	0.0352 (5)	
C5	0.77006 (18)	0.2441 (2)	0.47412 (14)	0.0371 (5)	
H5	0.7322	0.1807	0.5151	0.045*	
C6	0.88426 (18)	0.2553 (2)	0.48006 (14)	0.0378 (5)	
O2	0.93429 (13)	0.17546 (17)	0.54903 (10)	0.0498 (4)	
C7	1.05199 (19)	0.1787 (3)	0.55712 (17)	0.0562 (7)	
H7A	1.0827	0.1313	0.5043	0.084*	
H7B	1.0770	0.1186	0.6097	0.084*	
H7C	1.0773	0.2886	0.5630	0.084*	
C8	0.94020 (17)	0.3433 (2)	0.41983 (14)	0.0384 (5)	
H8	1.0187	0.3496	0.4239	0.046*	
C9	0.88069 (18)	0.4234 (2)	0.35258 (15)	0.0379 (5)	
O3	0.94471 (12)	0.50354 (18)	0.29569 (11)	0.0518 (5)	
C10	0.8906 (2)	0.5865 (3)	0.22476 (16)	0.0587 (7)	
H10A	0.8456	0.5124	0.1887	0.088*	
H10B	0.9459	0.6359	0.1880	0.088*	
H10C	0.8427	0.6685	0.2485	0.088*	
C11	0.76750 (17)	0.4184 (2)	0.34780 (14)	0.0359 (5)	
H11	0.7275	0.4778	0.3037	0.043*	
C12	0.34924 (17)	0.3117 (2)	0.35221 (13)	0.0341 (5)	
C13	0.26908 (18)	0.4123 (2)	0.38312 (14)	0.0392 (5)	
H13	0.2521	0.4093	0.4439	0.047*	
C14	0.21334 (18)	0.5168 (2)	0.32693 (15)	0.0427 (6)	
H14	0.1595	0.5868	0.3490	0.051*	
C15	0.23697 (18)	0.5181 (2)	0.23841 (15)	0.0426 (6)	
H15	0.1986	0.5893	0.1994	0.051*	
C16	0.31528 (18)	0.4178 (2)	0.20554 (14)	0.0399 (6)	
H16	0.3303	0.4193	0.1443	0.048*	
C17	0.37188 (17)	0.3148 (2)	0.26260 (14)	0.0351 (5)	
O4	0.44767 (12)	0.20606 (17)	0.23581 (9)	0.0440 (4)	
C18	0.4977 (2)	0.2339 (3)	0.15387 (16)	0.0613 (8)	
H18A	0.4430	0.2171	0.1051	0.092*	
H18B	0.5597	0.1605	0.1477	0.092*	
H18C	0.5249	0.3431	0.1522	0.092*	

### Atomic displacement parameters (Å^2^)

**Table:**

	*U*^11^	*U*^22^	*U*^33^	*U*^12^	*U*^13^	*U*^23^
C1	0.0341 (13)	0.0409 (13)	0.0364 (12)	−0.0028 (10)	0.0042 (10)	0.0045 (10)
O1	0.0405 (10)	0.0666 (11)	0.0637 (11)	−0.0077 (8)	0.0019 (8)	0.0290 (9)
C2	0.0336 (13)	0.0404 (13)	0.0357 (12)	0.0040 (10)	0.0005 (10)	0.0056 (10)
C3	0.0343 (13)	0.0366 (12)	0.0369 (12)	0.0016 (9)	0.0009 (10)	0.0009 (10)
C4	0.0357 (13)	0.0331 (11)	0.0371 (12)	0.0008 (10)	0.0051 (10)	−0.0023 (10)
C5	0.0377 (14)	0.0385 (12)	0.0350 (12)	0.0012 (10)	−0.0012 (10)	0.0049 (10)
C6	0.0405 (14)	0.0335 (12)	0.0388 (13)	0.0078 (10)	−0.0064 (11)	0.0005 (10)
O2	0.0446 (10)	0.0522 (10)	0.0517 (10)	0.0050 (8)	−0.0108 (8)	0.0071 (8)
C7	0.0429 (16)	0.0523 (15)	0.0717 (18)	0.0036 (12)	−0.0198 (13)	0.0017 (13)
C8	0.0283 (12)	0.0369 (12)	0.0497 (14)	0.0034 (10)	−0.0019 (10)	−0.0016 (11)
C9	0.0355 (13)	0.0327 (12)	0.0460 (14)	−0.0014 (10)	0.0087 (11)	0.0003 (10)
O3	0.0360 (10)	0.0558 (10)	0.0642 (11)	0.0011 (8)	0.0085 (8)	0.0177 (9)
C10	0.0510 (17)	0.0658 (17)	0.0600 (17)	0.0020 (13)	0.0098 (14)	0.0228 (14)
C11	0.0307 (13)	0.0341 (12)	0.0428 (13)	0.0019 (9)	0.0014 (10)	0.0018 (10)
C12	0.0317 (12)	0.0335 (11)	0.0374 (12)	−0.0039 (9)	0.0030 (10)	0.0025 (10)
C13	0.0375 (13)	0.0433 (13)	0.0370 (12)	−0.0041 (10)	0.0044 (10)	0.0006 (11)
C14	0.0348 (13)	0.0378 (13)	0.0555 (16)	0.0000 (10)	0.0036 (11)	−0.0028 (12)
C15	0.0350 (13)	0.0393 (13)	0.0530 (15)	−0.0023 (10)	−0.0050 (11)	0.0091 (11)
C16	0.0413 (14)	0.0417 (13)	0.0364 (13)	−0.0043 (11)	0.0002 (11)	0.0060 (11)
C17	0.0318 (12)	0.0348 (12)	0.0389 (13)	−0.0020 (10)	0.0040 (10)	−0.0005 (10)
O4	0.0471 (10)	0.0489 (9)	0.0363 (9)	0.0096 (7)	0.0061 (7)	0.0007 (7)
C18	0.0630 (19)	0.0699 (17)	0.0529 (16)	0.0118 (14)	0.0248 (14)	0.0074 (14)

### Geometric parameters (Å, º)

**Table:**

C1—O1	1.227 (2)	O3—C10	1.418 (3)
C1—C2	1.450 (3)	C10—H10A	0.9800
C1—C12	1.496 (3)	C10—H10B	0.9800
C2—C3	1.331 (3)	C10—H10C	0.9800
C2—H2	0.9500	C11—H11	0.9500
C3—C4	1.454 (3)	C12—C13	1.384 (3)
C3—H3	0.9500	C12—C17	1.392 (3)
C4—C5	1.390 (3)	C13—C14	1.381 (3)
C4—C11	1.398 (3)	C13—H13	0.9500
C5—C6	1.384 (3)	C14—C15	1.379 (3)
C5—H5	0.9500	C14—H14	0.9500
C6—O2	1.362 (2)	C15—C16	1.378 (3)
C6—C8	1.374 (3)	C15—H15	0.9500
O2—C7	1.424 (3)	C16—C17	1.386 (3)
C7—H7A	0.9800	C16—H16	0.9500
C7—H7B	0.9800	C17—O4	1.370 (2)
C7—H7C	0.9800	O4—C18	1.420 (2)
C8—C9	1.396 (3)	C18—H18A	0.9800
C8—H8	0.9500	C18—H18B	0.9800
C9—O3	1.361 (2)	C18—H18C	0.9800
C9—C11	1.368 (3)		
			
O1—C1—C2	120.4 (2)	O3—C10—H10B	109.5
O1—C1—C12	118.7 (2)	H10A—C10—H10B	109.5
C2—C1—C12	120.86 (18)	O3—C10—H10C	109.5
C3—C2—C1	124.2 (2)	H10A—C10—H10C	109.5
C3—C2—H2	117.9	H10B—C10—H10C	109.5
C1—C2—H2	117.9	C9—C11—C4	119.8 (2)
C2—C3—C4	127.2 (2)	C9—C11—H11	120.1
C2—C3—H3	116.4	C4—C11—H11	120.1
C4—C3—H3	116.4	C13—C12—C17	119.01 (19)
C5—C4—C11	119.6 (2)	C13—C12—C1	118.51 (18)
C5—C4—C3	122.21 (19)	C17—C12—C1	122.46 (18)
C11—C4—C3	118.11 (19)	C14—C13—C12	121.0 (2)
C6—C5—C4	119.7 (2)	C14—C13—H13	119.5
C6—C5—H5	120.1	C12—C13—H13	119.5
C4—C5—H5	120.1	C15—C14—C13	119.1 (2)
O2—C6—C8	124.0 (2)	C15—C14—H14	120.5
O2—C6—C5	115.25 (19)	C13—C14—H14	120.5
C8—C6—C5	120.7 (2)	C16—C15—C14	121.1 (2)
C6—O2—C7	117.86 (18)	C16—C15—H15	119.4
O2—C7—H7A	109.5	C14—C15—H15	119.4
O2—C7—H7B	109.5	C15—C16—C17	119.4 (2)
H7A—C7—H7B	109.5	C15—C16—H16	120.3
O2—C7—H7C	109.5	C17—C16—H16	120.3
H7A—C7—H7C	109.5	O4—C17—C16	123.80 (18)
H7B—C7—H7C	109.5	O4—C17—C12	115.80 (18)
C6—C8—C9	119.4 (2)	C16—C17—C12	120.32 (19)
C6—C8—H8	120.3	C17—O4—C18	117.45 (17)
C9—C8—H8	120.3	O4—C18—H18A	109.5
O3—C9—C11	125.1 (2)	O4—C18—H18B	109.5
O3—C9—C8	114.3 (2)	H18A—C18—H18B	109.5
C11—C9—C8	120.6 (2)	O4—C18—H18C	109.5
C9—O3—C10	117.80 (18)	H18A—C18—H18C	109.5
O3—C10—H10A	109.5	H18B—C18—H18C	109.5
			
O1—C1—C2—C3	−168.7 (2)	C5—C4—C11—C9	2.3 (3)
C12—C1—C2—C3	7.7 (3)	C3—C4—C11—C9	−175.44 (19)
C1—C2—C3—C4	179.76 (19)	O1—C1—C12—C13	54.6 (3)
C2—C3—C4—C5	−16.2 (3)	C2—C1—C12—C13	−121.9 (2)
C2—C3—C4—C11	161.5 (2)	O1—C1—C12—C17	−124.0 (2)
C11—C4—C5—C6	0.2 (3)	C2—C1—C12—C17	59.4 (3)
C3—C4—C5—C6	177.85 (19)	C17—C12—C13—C14	−1.3 (3)
C4—C5—C6—O2	177.74 (18)	C1—C12—C13—C14	−180.0 (2)
C4—C5—C6—C8	−1.7 (3)	C12—C13—C14—C15	1.2 (3)
C8—C6—O2—C7	−2.4 (3)	C13—C14—C15—C16	−0.3 (3)
C5—C6—O2—C7	178.19 (18)	C14—C15—C16—C17	−0.5 (3)
O2—C6—C8—C9	−178.67 (19)	C15—C16—C17—O4	177.12 (19)
C5—C6—C8—C9	0.7 (3)	C15—C16—C17—C12	0.5 (3)
C6—C8—C9—O3	−178.56 (18)	C13—C12—C17—O4	−176.49 (17)
C6—C8—C9—C11	1.9 (3)	C1—C12—C17—O4	2.1 (3)
C11—C9—O3—C10	−1.2 (3)	C13—C12—C17—C16	0.4 (3)
C8—C9—O3—C10	179.24 (19)	C1—C12—C17—C16	179.03 (19)
O3—C9—C11—C4	177.11 (18)	C16—C17—O4—C18	21.6 (3)
C8—C9—C11—C4	−3.4 (3)	C12—C17—O4—C18	−161.6 (2)

### Hydrogen-bond geometry (Å, º)

**Table:**

*D*—H···*A*	*D*—H	H···*A*	*D*···*A*	*D*—H···*A*
C2—H2···O1^i^	0.95	2.51	3.457 (3)	172

Symmetry code: (i) −*x*+1, −*y*, −*z*+1.
